# A Sketch (Analytical) of the History and Cure of Contagious Fever

**Published:** 1820-04-01

**Authors:** 


*A Sketch (Analytical) of the History and Cure of Contagious Fever.* By ROBERT JACKSON, M. D. 1 Vol. 8vo. pp. 284. London, 1819.

ALTHOUGH our Readers, as well as ourselves, have just cause to be tired of the subject of this volume; yet while fever continues to ravage on the vitals of the community, and men of experience to lay before their brethren the results of their observations, it is the duty of the Journalist to pourtray, and of the Reader to patiently audit, these records of passing events, in which all are more or less concerned.

Few Authors are more entitled to profound and respectful attention than the venerable Dr Jackson. The materials of his observations have been drawn from a boundless and fertile field of personal experience, during a period of little less than half a century; and that too with an active mind particularly directed to the investigation of febrile diseases. The sentiments of such a man carry with them a more than common degree of influence, since it is evident that they are not the results of first, and too often false impressions, but portraits corrected by time, reflection, and ample observation.

It is well known to our Readers, that Dr. Jackson's system of practice in the endemic fevers of unhealthy climes, was often stigmatized by our brethren at home as rash, or at least as ultra-depletory. However that may be, the volume now under review evinces no indications of rashness, but, on the contrary, forms a useful monitor to the ultra-phlebotomists of the present day In upholding the propriety of venesection in most of the complicated or aggravated forms of contagious fever, our Author wisely admits that blood-letting is by no means indispensably necessary in the simpler forms of this disease. He justly observes also, that "the power of the remedy is augmented by management;" since the abstraction of blood, merely as such, may be dangerous, unless executed with other accompaniments, as the warm bath, frictions, &c.

Dr. J. in his preface, throws out an opinion, which appears in unison with some continental theories, namely, that "the action of a febrile disease is principally manifested upon one system or series of parts; in the cutaneous expansions, internal or external." From this view, he presumes that fever is most speedily cured by those remedies which act directly and immediately upon the series of parts effected—"that is, the exhalents of the skin."

The greater part of the volume before us is occupied with a most interesting and instructive medical history of contagious fever, as it appeared on the Continent, in the British Isles, and in British Colonies, since the revolutionary war, among the armies, corps, or regiments, with which our author served. These histories are totally incapable of analysis, and must be attentively perused in the original by those who wish to see the facts and materials from which Dr. Jackson drew his doctrines and conclusions. We shall therefore pass at once to his "remarks on the prevailing epidemic."

> "Contagious fever, as it appears in fleets and armies, is almost always, if not always, an artificial disease, viz. the product of the accumulation of many persons under canvas in the field; in the narrow between decks of transport ships, or in damp and ill ventilated barracks. It has often a similar origin among masses of manufacturers, shut up in ill ventilated work-houses, or lodged in damp and ill ventilated cellars as places of dwelling. The disease, as originating from a common cause, is analogous in general form; it is modified more or less in appearance by circumstances of place or subject. A condition of atmosphere, artificially produced by aggregation and want of due ventilation, acts adversely on human health. It occasions a disease which, in the course of its proceeding, generates a material which is communicable to others, and which, thus communicated, propagates its kind through a series; in other words, becomes contagious." P. 147.

We need not observe that these are, and have long been, our own sentiments precisely. The testimony of a Jackson, on a matter of fact and observation like this, is, in itself, a host, and absolutely paramount. What may be the exact nature of the adventitious or febrific substances dispersed through the atmosphere, during epidemic constitutions, our Author does not pretend to explain. But it is evident that they are more concentrated at some points of the earth's surface than at others, and that they are modified by the revolutions of the seasons. The fever, which has been epidemic for a few years past in Great Britain and Ireland, Dr. Jackson pronounces to be "decidedly contagious;" though, in numerous instances, it arises from "a secret quality in the constitution of the atmosphere," at present unknown. He thinks the gastric form of fever, which is that most prevalent in Great Britain of late, is also that form which is most "readily convertible to contagious action." "The febrile act was principally manifested on serous secretions, or exhaling surfaces within the abdominal cavity; on the peritoneal surfaces, the interior of the alimentary canal, and, by continuity, on the exhaling surface of the skin."

"The function of the alimentary canal was almost always disturbed in one way or other; the expulsive power diminished in some with irksome sensations of desire; increased, at least irritated to exertion in others without effective purposes; the evacuations were changed in their nature, viz. dark, fœtid, often watery, dirty, copious, and of a sickly offensive smell, not feculent. Nausea and vomiting occurred occasionally; they were not often urgent; the stomach was sometimes inflated, the inflation accompanied with sensations of anxiety and distress; sometimes there was distress at stomach, without actual pain or distension." P. 151.

We cannot follow our observant author through the minute detail of symptoms, but pass on to other subjects. The duration of the disease, when left to itself, or feebly treated, was seldom under three weeks; though it was generally cut short, and "the patient restored to health in seven or eight days, where it was admitted to treatment at an early period, and treated with decision on a sound principle when submitted."

Our Author truly observes, that the character of the symptoms repeatedly changes, during the course of a single attack—"often so completely, that the form which succeeds has little resemblance, in superficial appearance, to that which preceded." In one case, for instance, the more prominent gastric symptoms are superceded by affection of the nervous system, intellectual and locomotive — in another, the morbid action is transferred to the thoracic organs—in many, to the coats of the intestines. This shews the absolute necessity of watching the morbid movements of fever, and being always ready to check them when in excess, instead of proceeding on any one routine principle of practice.

*Pathology.* As the contagious miasma which excites fever is invisible, so, in general, "the effect which the febrile act produces, is, in like manner, invisible."

"'If this be so, and it is reasonable and almost demonstrative that it is so, we are warranted to conclude that the changes which appear in organic structure after death, are changes contingent to the action of common disease, not the direct product of the action of the contagious process The action of the contagious cause, which appears to be directed to surfaces of serous, invisible secretion, cutaneous or other, is in some manner constrictive. It operates a change in the qualities of the secretion ; but it operates with so little violence, that no perceptible trace is left behind as a mark of the operation. Adhesion, purulent suppuration, congestion, &c. are foreign to its nature. But, though a visible change of structure does not belong to the operation of the cause of this form of disease, visible, and even considerable changes are observed not unfrequently in the bodies of those who die within the limit of the disease's action. They may, in so far as I have observed, be comprehended under the following heads. Where convulsion or other violence was the forerunner of actual death, the sinous veins within the head were generally turgid, literally engorged with black blood, more especially in relapse. Where the violence of the disease ceased suddenly, by what seemed to be an explosion of gangrene on an internal part, the peritoneal coat of the intestines was often in a state of black gangrene, without marks of local inflammation having preceded ; the liver, spleen, and sometimes the lungs, were filled with grumous blood. These appearances occurred frequently, as the first act of the disease in its relapsed form, in highly infected and ill ventilated hospitals, in cold, damp, and foggy weather; they were rare in the opposite circumstances. The contagious fever, when of a protracted course, often assumed the dysenteric form ; and in such form often terminated fatally. Where the termination was fatal, the inner coats of the alimentary canal were often loose and dissolved into bloody mucous, sometimes ulcerated and deeply corroded, oftener separated generally, almost through the whole extent; the exterior coat, black as if gangrened. Serous effusions into the ventricles in the brain, into the thorax and other cavities were observed occasionally. The substance of the brain was sometimes firm and full; sometimes flaccid and liquescent. Suppuration in some cases, adhesions in others, were observed in different places ; but these, and most others were contingent to the disease, not the effect of the action of the disease itself. The stomach and intestinal canal were sometimes pale, colourless, and inflated ; sometimes flaccid, withered, dry, — without moisture or unctuosity. This condition, which was not an unfrequent one, may be thought to be more nearly connected with the radical character of the disease than any of the others." P. 198.

*Treatment.* Our author observes, that the doctrines of debility and stimulation, which were almost universal in Great Britain, prior to the close of the last century, could not be said to lead to *inert* practice. The latter "produced changes, which, on some occasions, subverted the diseased course; it produced effects on others which accelerated or precipitated death." Without any fixed principle to guide them, some practitioners, assuming the existence of inflammation, congestion, or other derangement in the hepatic system, prescribed mercury in fever, and "the effect on the general issue of the disease was not unfrequently favourable." In other cases, the supposition of intestinal torpor suggested the idea of purgation, which was carried to a great extent by many, and, "in numerous instances, with benefit."

"From the decided benefit which followed the application of it in many cases, cold affusion was regarded, at one time, as a remedy of great promise: — it is useful; it is, however, like the two preceding, a remedy of circumstance only." P. 224.

Before our author enters on blood-letting, as a remedy in this disease, he naturally adverts to its pathology. Far from believing that fever has always a certain local habitation in the brain, he is not even satisfied that the "febrile act" is, in reality, a mode of inflammation.

"The act excited by the material cause of contagious fever, moves in channels of extreme minuteness. The human eye cannot discern the vessel, or measure its diameter; neither can the human mind venture, with safety, to form opinion as to the mode of change which takes place in the vessels action, whether inflammatory or otherwise. The product of inflammation is a visible, even a gross material. If inflammation be the act of the disease, the effect of the act might be expected to appear in dissection after death; yet, death takes place in many instances where no extraneous or diseased product can be discerned by the most penetrating sight. Inflammation is a forward or progressive act; and though its progress or its product cannot be discerned, it might still be supposed to exist, if experience did not furnish instances without number, where the primary action of the cause of fever actually is, at least is apparently, connected with conditions the reverse of inflammation; that is, with congestion, a condition of circulation in the veins urging to stagnation." P. 226.

While fever then may be said to be an act of the general system, still our veteran author believes that the impression of the febrific cause is made upon a susceptible surface, external or internal, whence it is conveyed, through channels not distinctly known, to the centre of the system — the seat of life and motion. From thence, he thinks, it is dispatched back to the series of parts first invaded "with a power generative of formal disease." "It receives form and becomes fever through the instrumentality of the sensorium: the sensorium is not the seat of the disease; and, I may add, that where the substance of the brain or its membranes are affected, the effect is contingent and secondary, as effects in other analogous structures." In these sentiments we agree with our experienced author.

"The principles assumed for the direction of the general cure of fever, whether resting on a supposition of debility, which implies stimulation as a remedy, or on a supposition of inflammation, which implies abstraction of blood and other processes of depletion, do not appear, as judged by experience, to give all the success to the practice that medical practice ought to have. The author has ventured to open a new ground. He speaks with humility; but he thinks he is entitled to speak with confidence, when he asserts, that more success is attainable from remedy, by considering the febrile act as an act changed from the action of health by an expression beyond just balance in force and activity at one time; by restriction, repression, or other cause which apparently diminishes the expression of open force at another, than by considering it as uniformly depressed, or uniformly excited. The diversity of condition is founded: the supposition of its existence has this advantage, if it have no other, that it obliges the physician to consider the case before he writes his prescription." P. 229.

This brings us to the position which we have so often laid down in this Journal, namely, that in fever, the balance of the circulation and excitement is broken; and that to restore it we must sometimes stimulate, sometimes deplete; nay, that we must sometimes stimulate one part of the system while we deplete another. We shall close this short analysis with the following extract.

"The remedies employed for the cure of contagious fever are of the same kind as those employed for the cure of the endemic; they are modified in application according to the circumstances of the case. Abstraction of blood, which, but a few years since, was viewed with abhorrence, even branded with the epithet of murder, is now considered as the main engine of successful treatment. The remedy was in a manner interdicted at the beginning of the war, 1793. I then employed it only rarely; that is, under the pressure of symptoms of extraordinay violence. In the beginning of the year 1796, chance gave me the opportunity of observing that, if it was not so necessary, it was not less safe in fevers of contagious origin, than in those which are distinctly endemic. Since that period I have employed it without fear of doing harm, generally with benefit; at least, with such benefit as to render the disease tractable to other remedies. Emetics have been employed by almost all practitioners at the early stages of contagious fever. They are of great value; judiciously managed, they often cut off the disease in its beginnings. I prefer emetics of severe operation, such as occasion sickness of long continuance. Purgatives of brisk operation, viz. jalap with calomel, emetic tartar or James's powder, and a small quantity of opium, had singular good effects in diminishing violence and danger, where the symptoms indicate mesenteric congestions, or where the disease is accompanied with intestinal torpor or irregular action in the bowels. Blisters applied to the temples, nape of the neck, and extended down the spine to the interval between the shoulders, are frequently employed at the earlier stages of this disease; they are employed with a marked good effect. Warm fomentations to the feet and legs, scrubbing of the skin with soap and brushes, warm bathing, followed by cold affusions, &c. often arrest the disease. Gestation in the open air, continued for a length of time, was a prescription of necessity, oftener than design; but it was mainly conducive, where employed, in confirming the health that was restored by the judicious and prompt application of the means now mentioned. The contagious action of the disease, I have reason to think, may be extinguished by the prompt and effectual application of the means stated; but, as complications arise not unfrequently during the course, especially at late periods, considerable modification is required to meet the circumstances of the case. P. 232.

We cannot enter on the didactic sections which make up the remainder of this valuable work, but must recommend them to the careful study of our brethren. Neither need we say any thing of the general merits of the publication. Dr. Jackson's name has, for many years, been a passport to professional respect and favour; and the work under review affords a convincing proof that hoary time has chilled nothing of that zeal and vigour of intellect so long and so successfully directed towards the advancement of medical science, and the mitigation of hnman afflictions!

> Stat sua cuique dies; breve et irreparabile tempus
> Omnibus est vitæ: sed famam extendere factis,
> Hoc virtutis opus.

